# Retrospective Observational Study on Diagnosis and Treatment Trends of DVT in Japan: Japanese Vein Study XXVI

**DOI:** 10.3400/avd.oa.25-00061

**Published:** 2025-08-14

**Authors:** Michihisa Umetsu, Takashi Yamaki, Tomohiro Ogawa, Toshiya Nishibe, Yasushi Shiraishi, Norikazu Yamada, Takashi Matsumoto, Tadashi Nomura, Atsushi Tabuchi, Yugo Yamashita, Hiroko Nemoto, Shinichi Hiromatsu, Makoto Mo

**Affiliations:** 1 Division of Vascular Surgery, Department of Surgery, Tohoku University Hospital, Sendai, Miyagi, Japan; 2 Department of Education and Support for Regional Medicine, Tohoku University Hospital, Sendai, Miyagi, Japan; 3 The Committee for the Survey of the Japanese Society of Phlebology, Tokyo, Japan; 4 Department of Plastic and Reconstructive Surgery, Tokyo Women’s Medical University Adachi Medical Center, Tokyo, Japan; 5 Department of Cardiovascular Surgery, Fukushima Dai-Ichi Hospital, Fukushima, Fukushima, Japan; 6 Department of Medical Informatics and Management, Hokkaido Information University, Ebetsu, Hokkaido, Japan; 7 Shiraishi Cardiovascular Clinic, Takamatsu, Kagawa, Japan; 8 Department of Cardiology, Kuwana City Medical Center, Kuwana, Mie, Japan; 9 Department of Vascular Surgery, Iizuka Hospital, Iizuka, Fukuoka, Japan; 10 Department of Plastic Surgery, Kobe University Graduate School of Medicine, Kobe, Hyogo, Japan; 11 Department of Cardiovascular Surgery, Kawasaki Medical School, Kurashiki, Okayama, Japan; 12 Department of Cardiovascular Medicine, Graduate School of Medicine, Kyoto University, Kyoto, Kyoto, Japan; 13 Department of Surgery, Yokohama City University, Yokohama, Kanagawa, Japan; 14 Department of Foot Care, Kurume University Medical Center, Kurume, Fukuoka, Japan; 15 Department of Cardiovascular Surgery, Yokohama Minami Kyosai Hospital, Yokohama, Kanagawa, Japan

**Keywords:** DVT, national survey, Japan, Japanese Vein Study

## Abstract

**Objectives:** The introduction of direct oral anticoagulants (DOACs) has significantly changed the management of deep vein thrombosis (DVT) in Japan. This study aimed to elucidate recent trend0s in the diagnosis and management of DVT following this shift.

**Methods:** This retrospective observational study involved 154 patients with acute and subacute DVT, and 96 patients with chronic or unknown-onset DVT, diagnosed between October 1 and 31, 2020, across 29 institutions affiliated with the Japanese Society of Phlebology. Data included patient demographics, diagnostic modalities, thrombus location, treatments, and clinical outcomes.

**Results:** The mean age was 70.0 years, and 57.8% of patients were female. Duplex ultrasonography was the predominant diagnostic modality (96.1%). DOACs were prescribed in 64.9% of patients, replacing warfarin and heparin. Compression therapy was used in 41.6% of patients. Soleal vein thrombosis was significantly more common in isolated distal DVT (right: 50.6% vs. 30.0%, p = 0.0082; left: 66.3% vs. 35.2%, p = 0.0001). Major bleeding occurred in 3.2% of patients. Post-thrombotic syndrome was observed in 0.6% of patients with acute/subacute DVT and 12.0% of those with chronic DVT patients.

**Conclusions:** Since the introduction of DOACs, DVT management in Japan has evolved considerably. Periodic multicenter surveys would be beneficial for evaluating long-term outcomes, treatment safety, and evolving clinical practices.

## Introduction

Lower extremity deep vein thrombosis (DVT) is a condition characterized by the formation of thrombi within the deep veins, resulting in symptoms such as edema, erythema, and pain. Risk factors including cancer, immobilization, postoperative status, and obesity are well-known contributors to the development of DVT.^1)^ In the acute phase, the thrombi can lead to pulmonary thromboembolism (PTE), while in the chronic phase, venous stasis may result in post-thrombotic syndrome (PTS), which occurs in approximately 20%–50% of patients with proximal DVT.^2,3)^

The main treatment for DVT is anticoagulant therapy. Compression therapy has also been shown to alleviate acute symptoms and potentially reduce the risk of PTS.^4–6)^ Thrombolytic therapy has been performed in selected cases; however, its use remains limited in Japan, not because of lack of indication but due to its limited adoption in clinical practice. More recently, thrombectomy devices and venous stents were approved in 2024 and are gradually being introduced at select institutions, offering alternative treatment options for patients with severe symptoms.^7,8)^ These treatments serve as supplementary therapies to standard anticoagulant therapy.

For many years, warfarin remained the principal anticoagulant in Japan because low-molecular-weight heparin (LMWH) had not been approved for therapeutic use. This led to discrepancies between Japanese and international practice, particularly in the management of cancer-associated thrombosis.^9,10)^ The introduction of direct oral anticoagulants (DOACs) in 2014 has helped close this gap, supported by growing evidence and updated treatment guidelines that now incorporate DOACs as standard therapy.^11–15)^

The Committee for the Survey of the Japanese Society of Phlebology has previously conducted nationwide surveys in 1997, 2004, and 2012 to document trends in DVT management. These surveys revealed a shift from venography to ultrasonography as the main diagnostic modality, as well as changes in the use of inferior vena cava (IVC) filters.^16–18)^

The present study aims to capture a contemporary, real-world snapshot of DVT management in Japan following the widespread adoption of DOACs. We conducted a retrospective, multicenter observational survey across 29 institutions to assess current diagnostic and treatment approaches, with the goal of informing future clinical strategies and identifying areas where practice may benefit from further standardization.

This study was organized by the Committee for the Survey of the Japanese Society of Phlebology, as part of its ongoing effort to monitor and report nationwide trends in DVT diagnosis and treatment.

## Materials and Methods

### Study design

This study is a retrospective, multicenter observational study designed to investigate current real-world practices in the diagnosis and treatment of DVT in Japan in the era of DOAC use and to compare these findings with data from a 2012 cohort. The study enrolled patients diagnosed with DVT between October 1 and October 31, 2020. No specific exclusion criteria were applied. Data collection was conducted between July and October 2024.

Although the main analysis focused on acute and subacute DVT, patients with chronic DVT were not excluded. A separate exploratory analysis was conducted for chronic DVT cases to reflect real-world practice and emerging treatment options. These were analyzed independently to avoid confounding the primary findings.

### Study population and data collection

Participating institutions were affiliated with the Japanese Society of Phlebology and voluntarily joined the survey. In total, 29 centers participated, including university hospitals, general hospitals, and smaller community-based facilities. This wide institutional spread reflects the decentralized nature of DVT care in Japan, where mid-sized or small hospitals account for a significant share of venous thromboembolism management. Patients diagnosed with DVT within the 1-month enrollment period were included. Investigators extracted data from medical records using a standardized Excel-based format. The collected information was compiled into a centralized database. Variables included patient demographics, date and method of diagnosis, diagnostic triggers, temporal classification of DVT, thrombus location, etiology, comorbidities, treatment, thrombus outcomes, bleeding complications, incidence of PTS, and mortality.

### Definitions

#### Diagnostic criteria and anatomical classification

DVT was diagnosed based on ultrasonographic evidence of non-compressibility on B-mode imaging and absent flow on Doppler, or an intraluminal filling defect on contrast-enhanced computed tomography (CT). Thrombosis was classified as proximal when involving the popliteal vein or above (even if calf veins were also involved), and distal when limited to the calf veins.

#### Temporal classification

DVT was temporally classified as acute when thrombus formation was estimated to have occurred within 2 weeks before diagnosis, subacute when it occurred between 2 and 6 weeks, and chronic when more than 6 weeks had elapsed.

#### Severity classification of PTE

PTE was categorized by severity into 4 types. The most severe form included cases presenting with cardiac arrest or circulatory collapse. Massive PTE was defined as hemodynamically unstable cases, characterized by shock or a sustained systolic blood pressure of less than 90 mmHg for at least 15 minutes, after excluding other causes such as arrhythmia, dehydration, or sepsis. Submassive PTE referred to hemodynamically stable patients with evidence of right heart strain detected on echocardiography. Non-massive PTE was defined as hemodynamically stable cases without signs of right heart strain on imaging.^19)^

#### Major bleeding

Major bleeding was defined according to the criteria of the International Society on Thrombosis and Haemostasis, which include fatal bleeding, symptomatic bleeding in critical areas (e.g., intracranial or retroperitoneal), a decrease in hemoglobin of ≥2 g/dL, or the need for transfusion of ≥2 units of whole blood or red blood cells.^20)^

#### Thrombus progression

Thrombus outcomes were categorized into 3 types: progression, defined as an increase in thrombus size compared to previous imaging; regression, indicating a partial reduction in thrombus burden; and resolution, referring to the complete disappearance of thrombus with restoration of venous flow.

#### PTS

PTS was diagnosed based on the presence of lower extremity symptoms with a Villalta score greater than 5 assessed at least 3 months after DVT onset. Severe PTS was defined as a Villalta score of 15 or higher.^19)^

### Statistical analysis

Categorical variables were reported as counts and percentages and compared using the chi-square or Fisher’s exact probability test. Continuous variables were summarized as mean ± standard deviation (SD) or median with interquartile range. Statistical significance was set at p <0.05 (2-sided). All analyses were conducted using JMP (version 17.2; SAS Institute, Cary, NC, USA).

## Results

### Patient characteristics and institutional distribution

A total of 250 patients were enrolled from 29 institutions. Among them, 96 patients (38.4%) were either classified as having chronic DVT or had DVT of uncertain onset, and were thus excluded from the primary analysis. The primary cohort thus consisted of 154 patients with acute or subacute DVT. Chronic DVT cases were analyzed separately at the end of this section.

The median age of patients in the primary cohort was 70.0 years (±14.8), and 57.8% were female. Recurrent DVT was observed in 7.8% of patients. Most cases (76.0%) were classified as acute DVT, and 24.0% as subacute. Diagnosis occurred during hospitalization in 60.4% of patients and in outpatient settings in 39.6%. University hospitals accounted for 37.7% of the cases, general hospitals for 57.8%, and clinics for 4.5% (**Table 1**).

**Table:** Table 1 Patient characteristics of acute and subacute DVT (N = 154)

	N	%
Characteristics		
Age, years (SD)	70	± 14.8
Female	89	(57.8)
Acute DVT	117	(76.0)
Subacute DVT	37	(24.0)
Recurrent DVT	12	(7.8)
Outpatient	59	(39.6)
Inpatient	90	(60.4)
Location		
Left	72	(47.1)
Right	39	(25.5)
Bilateral	42	(27.5)
Proximal DVT	71	(46.1)
Distal DVT	83	(53.9)
Diagnosis procedure		
Duplex ultrasonography	148	(96.1)
CT	43	(27.9)
MR	3	(1.9)
Only physical examination	0	(0.0)
Trigger for diagnosis		
Incidentally detected	8	(5.2)
Symptomatic	83	(53.9)
Preoperative screening	20	(13.0)
Postoperative screening	25	(16.2)
Elevated D-dimer	72	(46.8)
Embolic source evaluation	7	(4.5)

SD: standard deviation; DVT: deep vein thrombosis; CT: computed tomography; MR: magnetic resonance

### Diagnosis and etiology

Duplex ultrasonography was the most frequently used diagnostic modality (96.1%), followed by CT (27.9%). Diagnosis was prompted by clinical symptoms in 53.9% of cases, routine perioperative screening in 33.2%, elevated D-dimer levels in 46.8%, and incidental findings or embolic workup in a minority of cases.

The leading etiological factors were surgery (26.0%), immobilization (24.0%), and malignancy (23.4%). Idiopathic DVT accounted for 22.1%. Catheter-related thrombosis and hormone-related thromboses were rare. Thrombophilia screening was conducted in 27.3% of patients, with deficiencies in protein S, protein C, and antithrombin identified in a few (**Table 2**).

**Table:** Table 2 Etiology and comorbidities (N = 154)

	N	%
Etiology		
Surgery	40	(26.0)
Immobilization	37	(24.0)
Malignancy	36	(23.4)
Metastasis	12	(7.8)
Anticancer drugs	8	(5.2)
Idiopathic	34	(22.1)
Trauma	11	(7.1)
Dehydration	8	(5.2)
Cast immobilization	5	(3.2)
Pregnancy	5	(3.2)
Iliac compression	5	(3.2)
Catheter related	4	(2.6)
Oral contraceptive pill	1	(0.6)
Hormone replacement therapy	1	(0.6)
Prolonged travel	0	(0.0)
Comorbidities		
Chronic kidney disease	21	(13.6)
Varicose veins	13	(8.4)
Cerebrovascular disease	10	(6.5)
Autoimmune disease	8	(5.2)
Psychiatric disorder	7	(4.5)
Congestive heart failure	7	(4.5)
Liver disease	5	(3.2)
Inflammatory bowel disease	4	(2.6)
Antiphospholipid syndrome	3	(1.9)
Nephrotic syndrome	3	(1.9)
COVID-19	1	(0.6)
Thrombophilia screening		
Not performed	112	(72.7)
Protein S deficiency	4	(2.6)
Protein C deficiency	3	(1.9)
Antithrombin deficiency	2	(1.3)

COVID-19: coronavirus disease 2019

### Anatomic distribution and PTE

Distal DVT was slightly more common (53.9%) than proximal DVT (46.1%). Laterality was left-sided in 47.1%, right-sided in 25.5%, and bilateral in 27.5%. The most frequently affected veins were the soleal and popliteal veins. Iliac and IVC involvement was noted in 13.0% and 2.6%, respectively.

PTE was diagnosed in 26 patients (16.9%), including 1 with massive PTE, 6 with submassive PTE, and 19 with non-massive PTE (**Table 3**).

**Table:** Table 3 Anatomical location of thrombosis

	N	%	N	%
Concurrent PTE	26	(16.9)		
Massive PTE	1	(3.8)		
Submassive PTE	6	(23.1)		
Non-massive PTE	19	(73.1)		
IVC	4	(2.6)		
Proximal DVT	Rt		Lt	
Common iliac vein	4	(2.6)	5	(3.2)
External iliac vein	9	(5.8)	11	(7.1)
Common femoral vein	10	(6.5)	23	(14.9)
Femoral vein	16	(10.4)	36	(23.4)
Popliteal vein	22	(14.3)	37	(24.0)
Distal DVT	Rt		Lt	
Anterior tibial vein	1	(0.6)	5	(3.2)
Posterior tibial vein	16	(10.4)	19	(12.3)
Peroneal vein	22	(14.3)	28	(18.2)
Soleal vein	63	(40.9)	80	(51.9)
Gastrocnemius vein	5	(3.2)	13	(8.4)

IVC: inferior vena cava; PTE: pulmonary thromboembolism; DVT: deep vein thrombosis; Lt: left; Rt: right

### Treatment

DOACs were the mainstay of treatment, prescribed in 64.9% of cases—most commonly edoxaban, followed by apixaban and rivaroxaban. Warfarin and unfractionated heparin were less frequently used. Compression therapy was implemented in 41.6% of all patients and in 49.3% of those with symptoms.

Observation without anticoagulation or compression therapy was adopted in 13.0% of patients. Invasive treatments were rare; catheter-directed thrombolysis was performed in 1 case, and no patients underwent thrombectomy or stenting during the study period. IVC filters were placed in 8 patients; all retrievable filters were successfully removed (**Table 4**).

**Table:** Table 4 Treatment methods

	N	%
Observation	20	(13.0)
Compression therapy	64	(41.6)
Compression therapy for symptomatic DVT	42	(49.3)
DOAC	100	(64.9)
Edoxaban	46	(29.9)
Initial heparin	5	(10.9)
Apixaban	32	(20.8)
Loading dose	18	(56.3)
Rivaroxaban	22	(14.3)
Loading dose	13	(59.1)
Warfarin	11	(7.1)
Unfractionated heparin	19	(12.3)
Fondaparinux	0	(0.0)
Low-molecular-weight heparin	1	(0.6)
Urokinase	1	(0.6)
Tissue plasminogen activator	0	(0.0)
IVC filter	8	(5.2)
Temporary	2	
Retrievable	6	
Retrieved	6	
Catheter-directed thrombolysis, aspiration	1	(0.6)
Ballooning	0	(0.0)
Stenting	0	(0.0)
Surgical thrombectomy	0	(0.0)
Deep vein ligation	0	(0.0)
Bypass surgery	0	(0.0)
Arteriovenous shunt	0	(0.0)

DOAC: direct oral anticoagulants; IVC: inferior vena cava; DVT: deep vein thrombosis

### Clinical outcomes

No PTE-related deaths occurred during follow-up. The overall mortality rate was 13.0%, with equal proportions attributed to malignancy and other causes.

Thrombus regression was observed in 40.9%, and complete resolution in 23.4%. Thrombus progression occurred in 3 patients, and outcomes were unknown in 33.8%. The median duration of anticoagulation varied: 43.1% of patients received treatment for ≤6 months, while 34.3% were treated for >12 months. Patients with idiopathic DVT were more likely to receive prolonged therapy (p = 0.042), while no such trend was observed in cancer-associated DVT.

Major bleeding events occurred in 5 patients (3.2%), mostly gastrointestinal. PTS (Villalta score ≥5) was noted in 1 patient (0.6%) with acute or subacute DVT. Recurrent PTE occurred in 1 case (0.6%) after discontinuing anticoagulation (**Table 5**).

**Table:** Table 5 Clinical outcomes

	N	%
Death	20	(13.0)
Malignancy	10	
Others	10	
Outcome of thrombosis		
Extension	3	(1.9)
Regression	63	(40.9)
Complete resolution	36	(23.4)
Unknown	52	(33.8)
PTE after anticoagulation	1	(0.6)
Massive PTE	0	(0.0)
Submassive PTE	0	(0.0)
Non-massive PTE	1	(0.6)
Duration of anticoagulation		
<3 months	20	(19.6)
3 months	24	(23.5)
6 months	16	(15.7)
12 months	7	(6.9)
>12 months	35	(34.3)
PTS		
Villalta score >15	1	(0.6)
Major bleeding complication	5	(3.2)
Upper GI	1	
Lower GI	1	
Rectal cancer	1	
Hematuria	1	
Hemorrhagic infarction	1	

PTE: pulmonary thromboembolism; PTS: post-thrombotic syndrome; GI: gastrointestinal

### Comparison between proximal and distal DVT

Soleal vein involvement was significantly more common in distal DVT, while posterior tibial and gastrocnemius vein thrombosis were more frequent in proximal DVT.

Rivaroxaban was used significantly more often in proximal DVT (p = 0.0004). Although the duration of anticoagulation tended to be longer in proximal DVT, with 38.6% treated beyond 12 months compared to 28.9% in distal DVT, this difference was not statistically significant (**Table 6**).

**Table:** Table 6 Comparison between proximal and distal DVT

	Proximal DVT, N	%	Distal DVT, N	%	p Value
Rt common iliac vein	4	(5.6)			
Rt external iliac vein	9	(12.7)			
Rt common femoral vein	10	(14.1)			
Rt femoral vein	16	(22.5)			
Rt popliteal vein	22	(31.0)			
Rt anterior tibial vein	1	(1.4)	0	(0.0)	0.46
Rt posterior tibial vein	13	(18.3)	3	(3.6)	***0.0029***
Rt peroneal vein	11	(15.5)	11	(13.3)	0.69
Rt soleal vein	21	(30.0)	42	(50.6)	***0.0082***
Rt gastrocnemius vein	5	(7.0)	0	(0.0)	***0.019***
Lt common iliac vein	5	(7.0)			
Lt external iliac vein	11	(15.5)			
Lt common femoral vein	23	(32.4)			
Lt femoral vein	36	(50.7)			
Lt popliteal vein	37	(52.1)			
Lt anterior tibial vein	5	(7.0)	0	(0.0)	***0.019***
Lt posterior tibial vein	16	(22.5)	3	(3.6)	***0.0004***
Lt peroneal vein	20	(28.1)	8	(9.6)	***0.003***
Lt soleal vein	25	(35.2)	55	(66.3)	***0.0001***
Lt gastrocnemius vein	10	(14.0)	3	(3.6)	***0.038***

Statistical analysis was performed using the chi-square test or Fisher’s exact test, as appropriate.

DVT: deep vein thrombosis; Lt: left; Rt: right

### Chronic DVT

Among the 75 patients classified as having chronic thrombotic lesions, PTS with a Villalta score ≥5 was observed in 9 patients (12%), while severe PTS with a score ≥15 was noted in 4 patients (5.3%). Two patients underwent percutaneous balloon angioplasty with arterial stent placement (12 and 14 mm). Most others received conservative management: 34 patients underwent compression therapy, 23 were treated with DOACs, and 7 with warfarin. Twenty-six patients received no active treatment.

## Discussion

This study is the 1st multicenter survey on DVT management in Japan since the introduction of DOACs, conducted by the Committee for the Survey of the Japanese Society of Phlebology. By comparing our findings with those from the previous survey conducted in 2012, we evaluated changes in diagnostic and therapeutic approaches over the past decade and sought to identify current trends and challenges in real-world clinical practice.

The mean age of patients in this study was 70.0 years, with females comprising 57.8%, which is similar to the 2012 survey (mean age: 69.1 years). While surgery remained the most frequent provoking factor, the relative increase in cases associated with malignancy and immobilization may reflect heightened awareness among physicians of these non-surgical risks.

Duplex ultrasonography was the primary diagnostic modality in 96.1% of cases, representing a significant increase from 70% in 2004 and 87.7% in 2012. This trend highlights the maturation of ultrasound-based diagnosis, driven by technological improvements, the training of Clinical Vascular Technologists since 2006, and the publication of standardized protocols in 2018.^21)^ Nevertheless, the proportions of proximal versus distal DVT and the rate of concurrent PTE remained largely unchanged, indicating consistency in disease characteristics over time.

Treatment strategies showed substantial evolution. DOACs were prescribed in 64.9% of cases, largely replacing warfarin and unfractionated heparin. The practical advantages of DOACs, including the elimination of routine dose adjustments and their non-inferiority to LMWH in cancer patients, likely contributed to this shift.^22–24)^

In contrast, compression therapy utilization decreased from 89% in the previous survey to 41.6%. This reduction may be partly attributed to the increased detection of asymptomatic DVT, which accounted for 46% of patients. Additionally, 29% of patients were diagnosed through screening examinations. Changes in the medical specialties of society members may also have influenced this trend. Notably, the 2025 Japanese guidelines on compression therapy recommend compression as a Class IIa therapy for symptomatic DVT to improve acute-phase symptoms and reduce PTS.^4)^ Furthermore, the JCS/JPCPHS 2025 guideline on the management of pulmonary thromboembolism, deep venous thrombosis, and pulmonary hypertension also highlights the effectiveness of compression therapy in the acute and chronic phases.^15)^ Therefore, compression therapy for acute DVT may play an important therapeutic role alongside pharmacotherapy.

Regarding endovascular intervention, thrombectomy devices and venous stents became available in Japan in 2024 for severely symptomatic DVT cases, providing new treatment options. While only 1 case of PTS was recorded in this cohort of acute and subacute DVT, prior Japanese registry data (e.g., the COMMAND VTE Registry) reported a 13% incidence within 3 years.^25)^ In our chronic DVT subgroup, 9 patients (12%) had PTS, and 2 underwent angioplasty with large-diameter stents for chronic iliac vein occlusion. This aligns with current guidelines for the appropriate use of venous stents.^26)^

The anatomical analysis of distal DVT revealed a significantly higher prevalence of soleal vein thrombosis in isolated distal cases, in contrast to greater involvement of the posterior tibial and gastrocnemius veins in proximal DVT. These findings are physiologically plausible, as the soleal veins are located more peripherally and are prone to localized thrombus formation, whereas axial calf veins are more directly involved in propagation.^27)^ Given the differing natural histories of muscular and axial vein thrombosis, further research is warranted to develop risk-stratified treatment strategies, especially in cases of isolated soleal vein thrombosis.

Anticoagulation duration showed a bimodal pattern centered around 3 months and over 12 months, consistent with current guidelines recommending 3 months of treatment for provoked DVT and extended therapy for unprovoked or cancer-related DVT.^15)^ Although 4 major bleeding events were observed, none were fatal. Recent international studies suggest that reduced-dose extended therapy can maintain efficacy while lowering bleeding risk.^28–30)^ Although such regimens have not yet been approved in Japan, they may represent a valuable treatment option for patient populations in which safety is a priority, such as elderly patients or those at high risk of bleeding.

Several limitations should be acknowledged. First, the retrospective design may have led to incomplete data collection. Second, the study was limited to a 1-month enrollment period, despite extensive institutional involvement, which inherently restricted the sample size and the extent of longitudinal follow-up. Third, while this study aimed to provide a snapshot of real-world practice, participating institutions differed in scale and function, and this heterogeneity may limit direct comparability with previous surveys.

Despite these limitations, the findings of this study offer valuable cross-sectional insights into the evolving landscape of DVT management in Japan, particularly in the era of DOACs and guideline-driven care. These results may serve as a foundation for future prospective studies and policy-making aimed at optimizing treatment strategies for DVT.

## Conclusion

Since the introduction of DOACs in Japan, DVT management has transformed, alongside advances in diagnostic techniques. However, unresolved issues remain, including the need to raise awareness of the effectiveness of compression therapy and to establish individualized anticoagulation strategies, particularly for soleal vein thrombosis. Periodic nationwide surveys are essential to evaluate long-term outcomes and treatment safety.

## Declarations

### Ethical considerations

This study followed the Declaration of Helsinki (2013 revision) and the Ethical Guidelines for Medical and Biological Research Involving Human Subjects, issued by the Ministry of Health, Labor, and Welfare of Japan in April 2023. Given its retrospective nature using anonymized data, individual consent was waived, with opt-out disclosure via institutional websites. Approval was obtained from the Ethics Committee of Fukushima Daiichi Hospital (ID: 2407-02, Date: July 4, 2024).

### Acknowledgments

We deeply appreciate the efforts of all participating institutions for their valuable contributions despite the demands of daily clinical practice. The participating institutions were as follows: Hakodate Municipal Hospital, Tohoku University Hospital, Yamagata Saisei Hospital, Fukushima Medical University Hospital, Southern Tohoku Research Institute for Neuroscience, Japanese Red Cross Mito Hospital, Tsukuba Vascular Center, Tsuchiura Kyodo General Hospital, Saitama Medical University Hospital, Shin-yurigaoka General Hospital, Sakakibara Heart Institute, Toho University Ohashi Medical Center, Kyorin University Hospital, Ochanomizu Vascular and Vein Clinic, International University of Health and Welfare Narita Hospital, Yokohama Minami Kyosai Hospital, Mie University Hospital, Kuwana City Medical Center, Seiyu Memorial Hospital, Omi Medical Center, Ako Central Hospital, Kawasaki Medical School Hospital, Saiseikai Yamaguchi General Hospital, Japanese Red Cross Tokushima Hospital, Omuta City Hospital, Nagasaki University Hospital, Nagasaki Vascular Surgery Clinic, Kurume University Hospital, and Japanese Red Cross Kumamoto Hospital.

### Disclosure statement

YY received lecture fees and research funding from Daiichi Sankyo Co., Ltd., and research funding from Bayer Yakuhin Ltd. The other authors have no conflicts of interest to declare.

### Author contributions

Study conception: TY, MM

Data collection: MU, NY, AT, MM

Analysis: MU

Investigation: all authors

Manuscript preparation: MU

Critical review and revision: all authors

Final approval of the article: all authors

Accountability for all aspects of the work: all authors.

## References

[1] Nakamura M, Miyata T, Ozeki Y, et al. Current venous thromboembolism management and outcomes in Japan. Circ J 2014; 78: 708–17. 24401573 10.1253/circj.cj-13-0886

[2] Kahn SR, Shrier I, Julian JA, et al. Determinants and time course of the postthrombotic syndrome after acute deep venous thrombosis. Ann Intern Med 2008; 149: 698–707. 19017588 10.7326/0003-4819-149-10-200811180-00004

[3] Prandoni P, Lensing AW, Cogo A, et al. The long-term clinical course of acute deep venous thrombosis. Ann Intern Med 1996; 125: 1–7. 8644983 10.7326/0003-4819-125-1-199607010-00001

[4] Japanese Society of Phlebology. The 2025 Japanese Society of Phlebology (JSP) clinical practice guidelines for compression therapy in venous disease. Jpn J Phlebol 2025; 36: i–169. (in Japanese)

[5] Amin EE, Joore MA, Ten Cate H, et al. Clinical and economic impact of compression in the acute phase of deep vein thrombosis. J Thromb Haemost 2018; 16: 1555–63. 10.1111/jth.14163 29856509

[6] Aschwanden M, Jeanneret C, Koller MT, et al. Effect of prolonged treatment with compression stockings to prevent post-thrombotic sequelae: a randomized controlled trial. J Vasc Surg 2008; 47: 1015–21. 18372153 10.1016/j.jvs.2008.01.008

[7] Lopez R, DeMartino R, Fleming M, et al. Aspiration thrombectomy for acute iliofemoral or central deep venous thrombosis. J Vasc Surg Venous Lymphat Disord 2019; 7: 162–8. 30639411 10.1016/j.jvsv.2018.09.015

[8] Trujillo-Santos J, Demelo-Rodríguez P, Bravo de Laguna-Taboada A, et al. Optimizing venous stenting: consensus recommendations for enhanced management of lower extremity deep vein thrombosis. Semin Thromb Hemost 2024; 50: 883–93. 38733984 10.1055/s-0044-1786755

[9] Lee AY, Levine MN, Baker RI, et al. Low-molecular-weight heparin versus a coumarin for the prevention of recurrent venous thromboembolism in patients with cancer. N Engl J Med 2003; 349: 146–53. 12853587 10.1056/NEJMoa025313

[10] Lee AY, Kamphuisen PW, Meyer G, et al. Tinzaparin vs warfarin for treatment of acute venous thromboembolism in patients with active cancer: a randomized clinical trial. JAMA 2015; 314: 677–86. 26284719 10.1001/jama.2015.9243

[11] Kakkos SK, Gohel M, Baekgaard N, et al. Editor’s choice - European Society for Vascular Surgery (ESVS) 2021 clinical practice guidelines on the management of venous thrombosis. Eur J Vasc Endovasc Surg 2021; 61: 9–82. 33334670 10.1016/j.ejvs.2020.09.023

[12] Stevens SM, Woller SC, Kreuziger LB, et al. Antithrombotic therapy for VTE disease: second update of the CHEST guideline and expert panel report. Chest 2021; 160: e545–608. 34352278 10.1016/j.chest.2021.07.055

[13] Lyon AR, López-Fernández T, Couch LS, et al. 2022 ESC guidelines on cardio-oncology developed in collaboration with the European Hematology Association (EHA), the European Society for Therapeutic Radiology and Oncology (ESTRO) and the International Cardio-Oncology Society (IC-OS): developed by the task force on cardio-oncology of the European Society of Cardiology (ESC). Eur Heart J 2022; 43: 4229–361. 36017568 10.1093/eurheartj/ehac244

[14] Morán LO, Mateo FJP, Balanyà RP, et al. SEOM clinical guidelines on venous thromboembolism (VTE) and cancer (2023). Clin Transl Oncol 2024; 26: 2877–901. 39110395 10.1007/s12094-024-03605-2 PMC11467034

[15] Tamura Y. JCS/JPCPHS 2025 guideline on the management of pulmonary thromboembolism, deep venous thrombosis, and pulmonary hypertension. https://www.j-circ.or.jp/cms/wp-content/uploads/2025/03/JCS2025_Tamura.pdf (Accessed at 2025-3-30). (in Japanese)

[16] Hoshino S, Satokawa H. Deep vein thrombosis -Japanese vein study I. Jpn J Phlebol 1997; 8: 307–11. (in Japanese)

[17] Yamaki T, Hirai M, Ohta T, et al. The Japanese vein study: the 2nd survey of deep vein thrombosis. Jpn J Phlebol 2004; 15: 79–85. (in Japanese)

[18] Satokawa H, Yamaki T, Iwata H, et al. The survey of deep vein thrombosis and venous thromboembolism prevention: Japanese vein study XII. Jpn J Phlebol 2012; 23: 271–81. (in Japanese)

[19] Ito M. Guidelines for diagnosis, treatment and prevention of pulmonary thromboembolism and deep vein thrombosis (JCS 2017). https://j-circ.or.jp/old/guideline/pdf/JCS2017_ito_h.pdf (Accessed at 2025-2-25). 10.1253/circj.cj-88-0010 21441695

[20] Schulman S, Kearon C. Definition of major bleeding in clinical investigations of antihemostatic medicinal products in non-surgical patients. J Thromb Haemost 2005; 3: 692–4. 15842354 10.1111/j.1538-7836.2005.01204.x

[21] Working Group on Venous Ultrasonography. Standardized ultrasound assessment of deep vein thrombosis and lower extremity varicose veins. Jpn J Phlebol 2018; 29: 363–94. (in Japanese)

[22] Raskob GE, van Es N, Verhamme P, et al. Edoxaban for the treatment of cancer-associated venous thromboembolism. N Engl J Med 2018; 378: 615–24. 29231094 10.1056/NEJMoa1711948

[23] Agnelli G, Becattini C, Meyer G, et al. Apixaban for the treatment of venous thromboembolism associated with cancer. N Engl J Med 2020; 382: 1599–607. 32223112 10.1056/NEJMoa1915103

[24] Young AM, Marshall A, Thirlwall J, et al. Comparison of an oral factor Xa inhibitor with low molecular weight heparin in patients with cancer with venous thromboembolism: results of a randomized trial (SELECT-D). J Clin Oncol 2018; 36: 2017–23. 29746227 10.1200/JCO.2018.78.8034

[25] Nishimoto Y, Yamashita Y, Morimoto T, et al. Risk factors for post-thrombotic syndrome in patients with deep vein thrombosis: from the COMMAND VTE registry. Heart Vessels 2019; 34: 669–77. 30293163 10.1007/s00380-018-1277-3

[26] Japanese Society of Phlebology. Appropriate use criteria for venous stents in Japan. https://js-phlebology.jp/?page_id=3099 (Accessed at 2025-4-12). (in Japanese)

[27] Wang C, Shi C, Guo R, et al. Comparison of clinical outcomes among patients with isolated axial vs muscular calf vein thrombosis: a systematic review and meta-analysis. J Vasc Surg Venous Lymphat Disord 2024; 12: 101727. 38043681 10.1016/j.jvsv.2023.101727 PMC11523313

[28] Agnelli G, Buller HR, Cohen A, et al. Apixaban for extended treatment of venous thromboembolism. N Engl J Med 2013; 368: 699–708. 23216615 10.1056/NEJMoa1207541

[29] Weitz JI, Lensing AWA, Prins MH, et al. Rivaroxaban or aspirin for extended treatment of venous thromboembolism. N Engl J Med 2017; 376: 1211–22. 28316279 10.1056/NEJMoa1700518

[30] Couturaud F, Schmidt J, Sanchez O, et al. Extended treatment of venous thromboembolism with reduced-dose versus full-dose direct oral anticoagulants in patients at high risk of recurrence: a non-inferiority, multicentre, randomised, open-label, blinded endpoint trial. Lancet 2025; 405: 725–35. 40023651 10.1016/S0140-6736(24)02842-3

